# Assessment and Rating of Motor Cerebellar Ataxias With the Kinect v2 Depth Sensor: Extending Our Appraisal

**DOI:** 10.3389/fneur.2020.00179

**Published:** 2020-03-11

**Authors:** Takeru Honda, Hiroshi Mitoma, Hirotaka Yoshida, Kyota Bando, Hiroo Terashi, Takeshi Taguchi, Yohane Miyata, Satoko Kumada, Takashi Hanakawa, Hitoshi Aizawa, Shiro Yano, Toshiyuki Kondo, Hidehiro Mizusawa, Mario Manto, Shinji Kakei

**Affiliations:** ^1^Movement Disorders Project, Tokyo Metropolitan Institute of Medical Science, Tokyo, Japan; ^2^Department of Advanced Neuroimaging, Integrative Brain Imaging Center (IBIC), National Center of Neurology and Psychiatry, Tokyo, Japan; ^3^Medical Education Promotion Center, Tokyo Medical University, Tokyo, Japan; ^4^Department of Computer and Information Sciences, Tokyo University of Agriculture and Technology, Tokyo, Japan; ^5^Department of Neurology, Tokyo Medical University, Tokyo, Japan; ^6^Department of Neuropediatrics, Tokyo Metropolitan Neurological Hospital, Tokyo, Japan; ^7^National Center Hospital, National Center of Neurology and Psychiatry, Tokyo, Japan; ^8^Department of Neurosciences, University of Mons, Mons, Belgium

**Keywords:** ataxia, motor control, cerebellar degeneration, SARA, ICARS, depth sensor

## Abstract

Current assessment of patients with cerebellar disorders is based on conventional neurological examination that is dependent on subjective judgements. Quantitative measurement of cerebellar ataxias (CAs) is essential for assessment of evidence-based treatments and the monitoring of the progress or recovery of diseases. It may provide us a useful tool to navigate future treatments for ataxia. We developed a Kinect v2. sensor system with a novel algorithm to measure and evaluate movements for two tests of Scale for the Assessment and Rating of Ataxia (SARA): the nose-finger test and gait. For the nose-finger test, we evaluated and compared accuracy, regularities and smoothness in the movements of the index finger and the proximal limbs between cerebellar patients and control subjects. For the task of walking, we evaluated and compared stability between the two groups. The precision of the system for evaluation of movements was smaller than 2 mm. For the nose-finger test, the mildly affected patients tended to show more instability than the control subjects. For a severely affected patient, our system quantified the instability of movements of the index finger using kinematic parameters, such as fluctuations and average speed. The average speed appears to be the most sensitive parameter that contrasts between patients with CAs and control subjects. Furthermore, our system also detected the adventitious movements of more proximal body parts, such as the elbow, shoulder and head. Assessment of walking was possible only in patients with mild CAs. They demonstrated large sways and compensatory wide stances. These parameters appeared to show higher accuracy than SARA. This examiner-independent device measures movements of the points of interest of SARA more accurately than eye and further provides additional information about the ataxic movements (e.g., the adventitious movements of the elbow, shoulder and head in the nose-finger test and the wide-based walking with large oscillation in the gait task), which is out of the scope of SARA. Our new system enables more accurate scoring of SARA and further provides additional information that is not currently evaluated with SARA. Therefore, it provides an easier, more accurate and more systematic description of CAs.

## Introduction

Disorder of the cerebellum such as cerebellar degeneration causes disorganizations in limb and trunk movements (1). The severity of motor symptoms in cerebellar ataxias (CAs) is currently quantified using various clinical scales, such as the International cooperative ataxia rating scale (ICARS) (2) or the Scale for the assessment and rating of ataxia (SARA) (3). For example, the SARA evaluates the degree of CAs by measuring the following tasks: (i) the task of standing/sitting/walking, which examines instability and irregularity in lower limbs and trunk, (ii) the task of finger to nose/heel to knee test and the task of pursuit of an index, both of which quantify dysmetria in upper/lower limb, and (iii) the task of forearm pronation and supination, which analyzes adiadochokinesis (3).

These clinical scores have disadvantages. First, it is not sensitive enough to quantify subtle changes of cerebellar ataxia. For instance, SARA scores change on average <1 point per year in SCA6 patients (4). Second, it is difficult to avoid examiner-dependent variations in overall scores, especially among non-expert examiners, since assessment of scores employed in these scales depend on subjective judgement by each examiner on the order of several centimeters.

One way to make current SARA much more sensitive and accurate may be introducing digital motion analysis for evaluation of SARA. Several objective and quantitative tests have been developed for evaluation of ataxia (e.g., (5–8) for forelimb movement, (9–11) for gait, or (12, 13) using Kinect]. However, these studies were not specifically designed to improve the accuracy of SARA itself.

This study was designed to develop a device which improves accuracy of SARA as a whole by one order (i.e., on the order of millimeters). More specifically, we intended to demonstrate improved accuracy, precision, and the efficacy of our device in capturing ataxic movements during two tasks of SARA; the nose-finger test representing upper limb movements and walking representing movements of lower limbs and trunk. We also intended to show comprehensively the quantitative evaluation of ataxic movement for SARA tests using our devise.

## Materials and Methods

### Participants

Five control subjects with no history of neurological abnormalities [two males (74 and 78 years old) and three females (41, 63, and 68 years old), all right handed] and five patients with cerebellar degeneration [three males (46, 62, and 73 years old) and two females (62 and 68 years old), all right handed] participated in the study. The mean age of the control subjects was 64.8 ± 14.5 years and that of the patients was 62.2 ± 10.2 years (*p* = 0.726, *t*-test). One patient had spinocerebellar ataxia type 6 (SCA6) as confirmed by genetic testing, and two patients had sporadic cortical cerebellar atrophy (CCA) (14, 15). Two multiple system atrophy (MSA-C: cerebellar variant) patients (16) exhibited clear signs of cerebellar disease. The same person rated the SARA. The experimental procedure was approved by the Ethics Committee of Tokyo Medical University (2017-035), National Center of Neurology and Psychiatry (A2018-104) and Tokyo Metropolitan Institute of Medical Science (18–41).

### Apparatus and SARA Task

**Figure 1:**
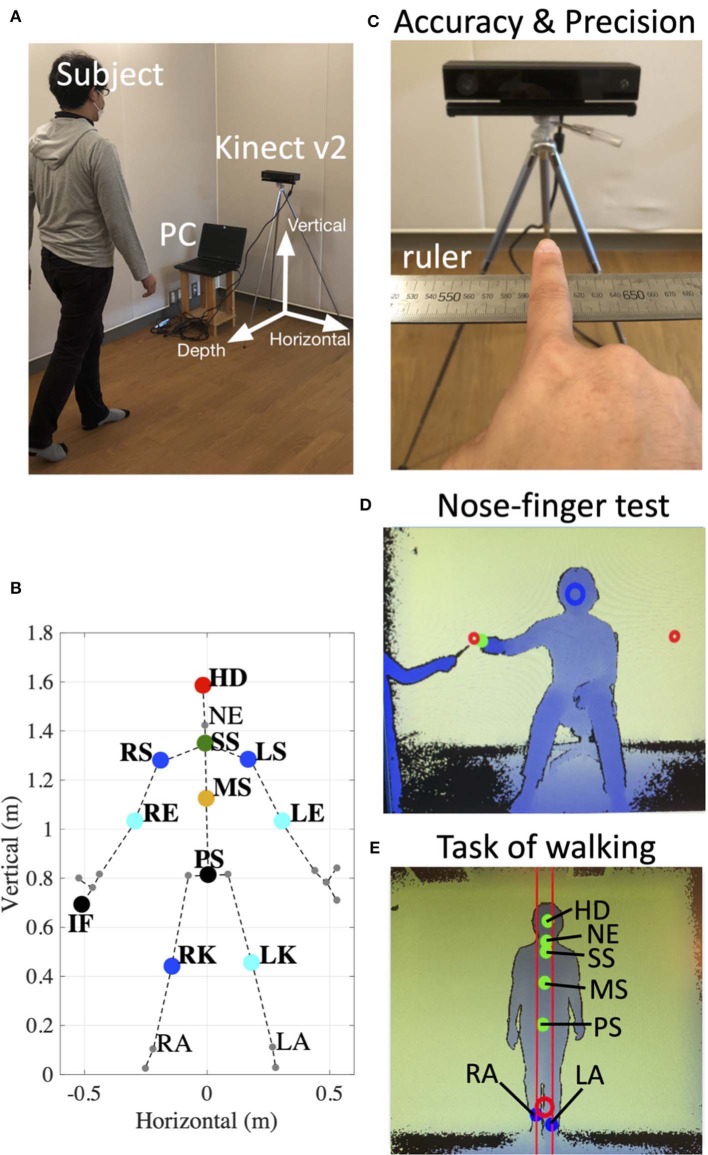
Experimental setup. **(A)** Arrangement of the device. Informed consent was obtained from the individual for the publication of this image. **(B)** 25 reference points (large dots and small dots) detected by the Kinect v2. In the present study, we focus on the markers of the right index finger (IF), right shoulder (RS), left shoulder (LS), right elbow (RE), left elbow (LE), right knee (RK), left knee (LK), head (HD), middle point of RS and LS on the spine (SS), pelvis on the spine (PS), a middle point of SS and PS (MS), neck (NE), right ankle (RA), and left ankle (LA). **(C)** The setup to evaluate accuracy and precision of the Kinect v2. The position of subject's IF on the ruler was compared with values obtained from the device. **(D)** An image obtained from depth data in the nose-finger test. The green dot indicates the position of subject's IF. The red circles show target positions. The subject was instructed to keep his/her nose within the blue circle. **(E)** An image obtained from depth data in the task of walking. The dots show the positions of HD, NE, SS, MS, and PS, RA, and LA. The subject was instructed to hold a center position of his/her RA and LA within the red circle.

We developed a device that consists of a windows10 PC and a Kinect v2. sensor (Microsoft Co.) (Figure 1A). We also developed a dedicated software using the Kinect for Windows SDK 2.0 and Visual Studio 2015 (Microsoft Co.). In Windows SDK, the classifier estimates three-dimensional positions of body parts from each depth image (17). Our device recorded three-dimensional (horizontal, vertical and depth as shown in Figure 1A) positions of subjects' right index finger (IF), right shoulder (RS), left shoulder (LS), right elbow (RE), left elbow (LE), right knee (RK), left knee (LK), head (HD), and Trunk (Tr) consisting of a middle point of their left and right shoulders on their spine (SS), pelvis on their spine (PS), a middle point of SS and PS (MS), neck (NE), right ankle (RA), and left ankle (LA). These positions were collected from the Kinect v2. sensor at every 33 ms (30 Hz) (Figure 1B).

**Figure 2:**
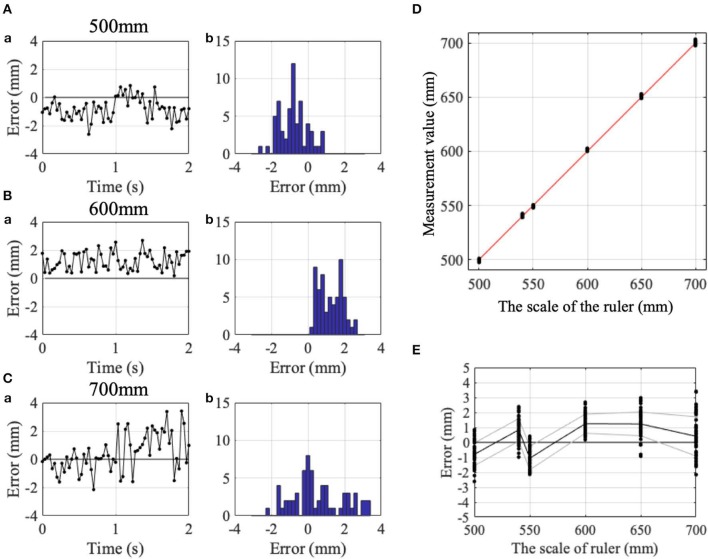
Accuracy and precision of the Kinect v2. (**A**a) Change in detection errors with time and (**A**b) their histogram, while IF was kept at the 500 mm point on the ruler. (**B**a) Similar change in detection errors with time and (**B**b) their histogram at the 600 mm point on the ruler. (**C**a) Similar change in detection errors with time and (**C**b) their histogram at the 700 mm point on the ruler. **(D)** Comparison of the read of the ruler (abscissa) and the measurement value obtained from the Kinect v2 (ordinate). Red dashed line indicates the identity line. **(E)** Error between the measurement value and the read of the ruler. Black and gray lines indicate means and standard deviations. Positive and negative values showed horizontal positions rightward and leftward as shown in, respectively.

To evaluate accuracy and stability of measurements of the Kinect v2, three control subjects sitting in front of the Kinect v2. were asked to keep their index fingers on six fixed points on a ruler (500, 540, 550, 600, 650, and 700 mm) immobilized for 2 s (Figures 1C, 2). We obtained three-dimensional data of each position of the index fingers of three control subjects from the Kinect v2.

For the nose-finger test, the subjects sat in front of the Kinect v2. and moved their index finger from their nose to the top of a pointer that the examiner held in front of them (Figure 1D). The examiner adjusted the examinee's position so that his/her nose is within the blue circle in the monitor (Figure 1D). The position of the tip of the index finger was displayed as the cursor on the monitor (green dot in Figure 1D). The nose-finger test was repeated 10 times for each participant.

For the walking task, the examiner adjusted the examinee's trunk (SS, MS, and PS) within two red lines on the monitor (Figure 1E). We analyzed movements of six reference points (HD, NE, SS, MS, PS, RK, and LK) of subjects during 1.5 m walking (3–1.5 m from the Kinect v2. sensor) in normal gait and in tandem walk (Figure 1E). The positions of HD, NE, SS, MS, PS, RA, and LA were displayed on the monitor (green dots in Figure 1E).

### Data Analysis and Statistics

We analyzed data obtained from the Kinect v2. using MATLAB 2018b. The horizontal, vertical and depth axes for the measurement are shown in Figure 1A. The positive value for each axis indicates rightward, upward and backward movement of the subject.

To evaluate accuracy and stability of measurements of the Kinect v2, we calculated mean positional errors and mean standard deviations of the positional errors. The positional errors indicate the difference between measurement value from the Kinect v2. and the true value (i.e., read of scale). The mean standard deviations of the positional errors were calculated for each of the six points on the ruler.

To compare variance of touching points in the nose-finger test between control subjects and cerebellar patients, we calculated Ansari-Bradley test in Figure 4.

To compare various movement parameters of nose-finger test between the patients and the controls, we used a Mann–Whitney *U*-test (Figure 5). To compare head sway and inter-knee distance of the task of walking between the controls and the patients, we also used a Mann–Whitney *U*-test (Figure 8). In addition, Spearman's rank correlations were calculated for comparison of various parameters of the nose-finger test and the task of walking with SARA scores (Figures 5, 8).

## Results

### Accuracy of Measurement With Kinect v2

To determine the measurement accuracy of Kinect v2, we measured position of participant's index finger for 2 s at 30 Hz, while the subjects pointed his/her IF to Kinect v2. without movements (Figure 1C). Figures 2A–C demonstrate examples of temporal patterns of measurement for pointing at 500, 600, and 700 mm, respectively. Although instantaneous deviation of values sometimes exceeded more than 2 mm for each point (Figures 2A–C), time averages of measurement was stable for the three pointing.

Black dots in Figure 2D represent relationship between finger positions on the ruler and measurement values obtained with Kinect v2. at each point. It should be noted that the relationship is almost linear (red line in Figure 2D). Measurement values with Kinect v2 are quite proportional to the scale of the ruler. The mean positional errors (i.e., accuracy) for the six points on the ruler in the three control subjects were 0.310 ± 1.008 (Figure 2), −0.203 ± 1.512, and −0.082±1.037 mm, respectively. The mean standard deviations of the positional errors (i.e., precision) in the three control subjects were 0.838 ± 0.238 (Figure 2), 0.514 ± 0.203 and 0.629 ± 0.332 mm, respectively. The mean positional error and mean standard of the positional errors deviations for the three control subjects are 0.009 ± 0.268 mm (*n* = 3) and 0.661 ± 0.164 mm (*n* = 3), respectively. It should be noted that standard deviations of the mean positional errors were 1.008, 1.512, and 1.037 mm, for the three subjects, for the measurement of the stationary finger. It is expected that the error increases for moving body parts because fewer number of samples are available for each position for the fixed sampling rate (i.e., 30 Hz). Overall, accuracy of the measurement with Kinect v2 are estimated to be <2 mm for slower movements employed in SARA.

### Nose-Finger Test

**Figure 3:**
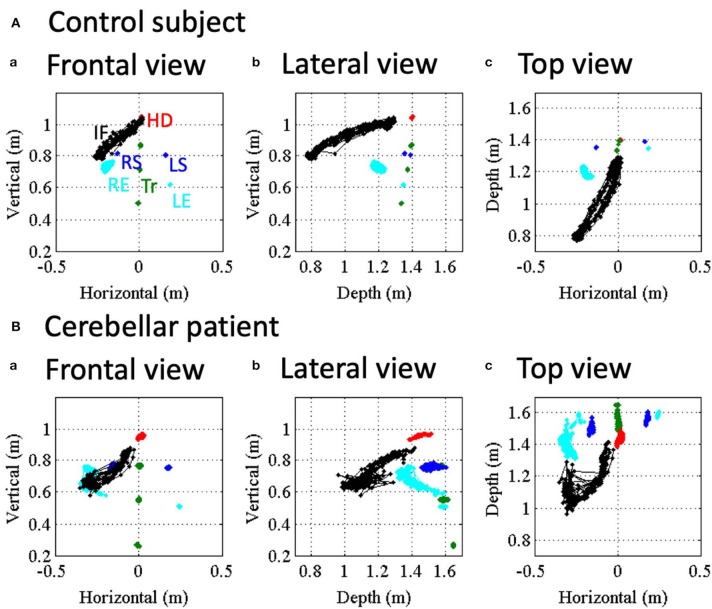
Movement kinematics of reference points in the nose-finger test for a control **(A)** and an ataxic patient. **(B)** Red, blue, cyan and black dots show positions of HD, shoulders (RS/LS), elbows (RE/LE) and IF, respectively. Trunk (Tr) consists of SS, PS and MS (green dots). (a–c) Correspond to frontal view, lateral view and top view, respectively.

Figure 3 shows movements of various body parts during the nose-finger test for a control subject and a patient with CCA. Positions of index finger (IF, black dots), head (HD, red dots), right and left shoulders (RS and LS, blue dots), right and left elbows (RE and LE (cyan dots) and trunk (TR, green dots) are displayed with different colors for a control subject and a patient with MSA-C. The trajectories of these reference points were superimposed during the repeated 10 strokes of IF movements. In the control subject, the trajectories of IF were rather constant throughout the repeated trials, i.e., she correctly placed her IF on both the nose and the target with approximately same trajectories (Figure 3A). On the other hand, other body parts except the RE were stable. Thus, the control subject made movements of IF mainly by using shoulder and elbow joints, with the other joints immobilized. In contrast, in the ataxic patient (Figure 3B), the positions of her IF were scattered during the repeated trials. It should be noted that IF deviated abruptly from the target (Figure 3B), which reflects terminal (kinetic) tremor. On the other hand, positions of other markers demonstrated much more instability of the proximal arm and the trunk than the control subject. In other words, the ataxic movement of IF is composed of fluctuation of trunk as well as arm itself.

**Figure 4:**
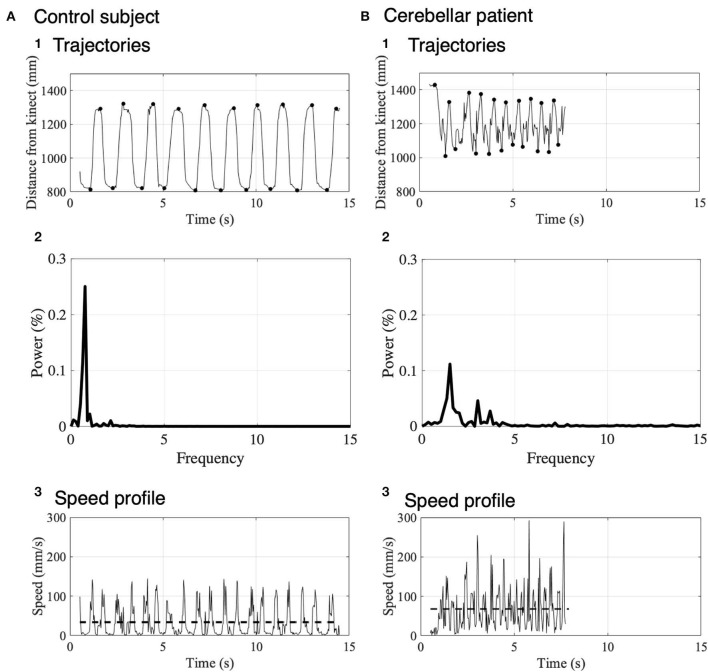
Movement kinematics of IF in the nose-finger test for a control subject **(A)** and a patient **(B)**. **(A**1**)** Trajectory of IF measured as the distance from the Kinect v2 to IF in a control subject. Black dots show the local maxima and local minima of the distance. **(A**2**)** Spectrum analysis of trajectories of IF shown in **(A**1**)**. Note that the power is normalized. **(A**3**)** Speed profile represented by the difference of the trajectory in the control subject. The dashed lines indicate the average speeds. **(B)** Trajectory **(B**1**)**, Spectrum analysis of trajectories of IF **(B**2**)** and velocity profile **(B**3**)** in a cerebellar patient. The same conventions as in **(A)**.

Figure 4 shows distance of IF from Kinect v2 for the control subject (Figure 4A1) and the patient (Figure 4B1). The control subject showed a regular and smooth IF-movement, while the patient demonstrated irregular fluctuations, suggesting ataxic movements. To evaluate stability of reaching movement to the nose and the target, we measured local maxima and local minima of IF-movements for the control subject (black dots in Figure 4A1) and the patient (black dots in Figure 4B1). The local maxima and the local minima correspond to positions of touches on the nose and touches on the target, respectively. For this particular control subject, average positions of nose touch and target touch were 1306.70 ± 12.64 mm (*n* = 10 repeats) and 814.14 ± 5.66 mm (*n* = 10 repeats), respectively. On the other hand, for this particular patient, those of the nose touch and target touch were 1352.21 ± 33.73 mm (*n* = 10 repeats) and 1043.36 ± 22.93 mm (*n* = 10 repeats), respectively. It should be emphasized that the variance of these distributions was significantly larger for the patient than the control in the nose touch (*p* = 0.028, Ansari–Bradley test). Additionally, to characterize cerebellar ataxia, we performed a spectral analysis of trajectories shown in Figures 4A1, B1. In the control subject, there was a large peak at 0.7 Hz (Figure 4A2) corresponding to the main frequency of the IF movements (Figure 4A1). In contrast, in the patient, there were three peaks at 1.5, 3.0, and 3.7 Hz (Figure 4B2). The width of the primary peak of the patient at 1.5 Hz is larger than that of the control, suggesting that the main frequency of the movement fluctuates in cerebellar ataxia. Moreover, there were two additional peaks at higher frequency (3.0 and 3.7 Hz) than the primary peak, reflecting complex irregularity of the ataxic movements (Figure 4B1). Next, we calculated velocity profiles of the movement of IF for the control (Figure 4A3) and the patient (Figure 4B3). For the patient, velocity peaks were higher and more variable than the control, suggesting more irregular movement of IF.

**Figure 5:**
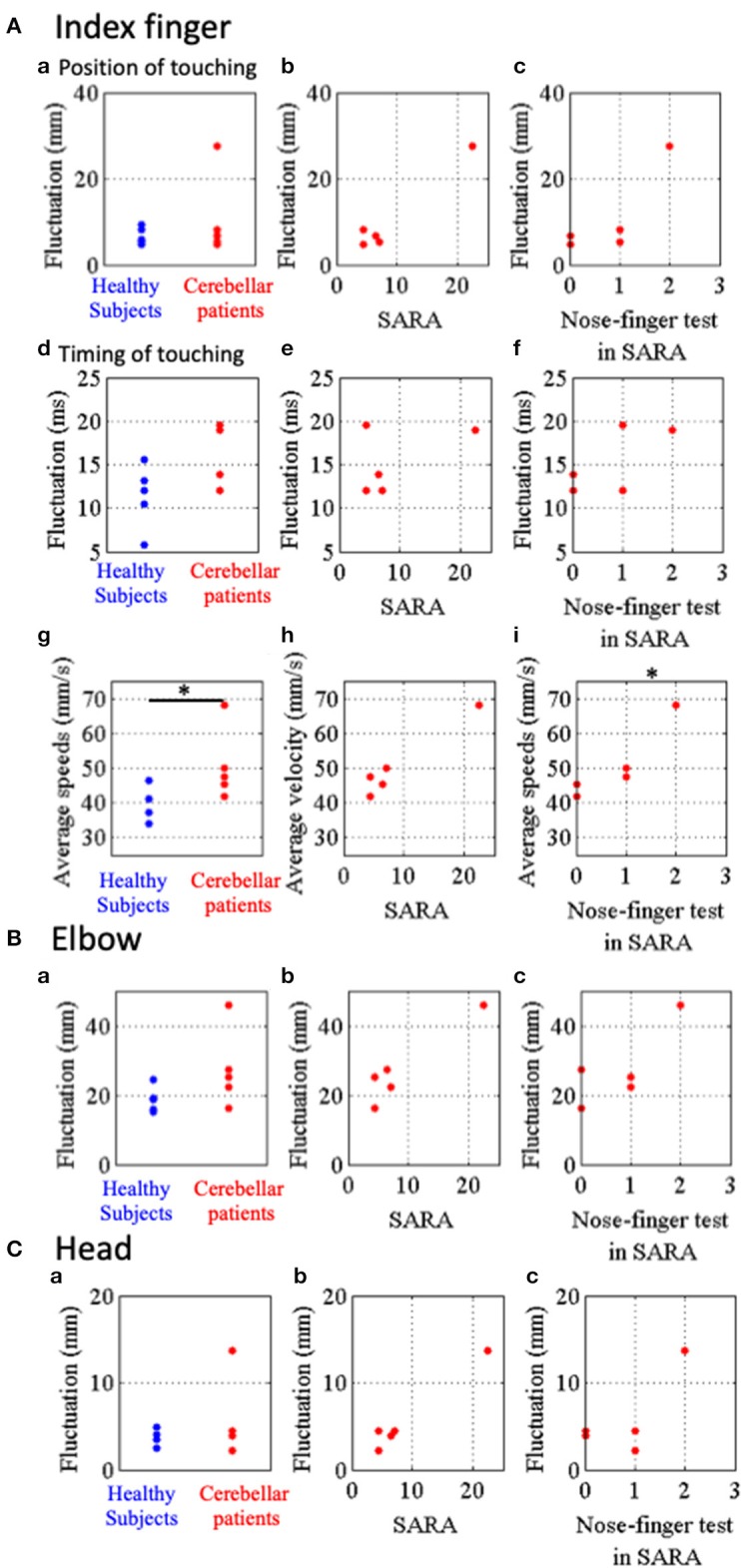
Parameters representing stability of movements of IF, RE and HD in the nose-finger test. **(A)** Parameters for IF movements. (a) Blue and red dots show fluctuations of IF movements of control subjects and cerebellar patients, respectively. Relationship between the parameters of fluctuation and SARA score **(B)** or score of the nose-finger test in SARA **(C)**. (d–f) Timing of touching. (d) Blue and red dots show temporal fluctuations of IF movements of control subjects and cerebellar patients, respectively. (g–i) Average speeds of IF movements of control subjects (blue dots) and cerebellar patients (red dots). **(B)** Fluctuations of RE movements of control subjects (blue dots) and cerebellar patients (red dots). **(C)** Fluctuations of HD movements of control subjects (blue dots) and cerebellar patients (red dots). *p < 0.05 by Mann–Whitney *U*-test (g), and *p < 0.05 by Spearman's rank correlation coefficient (otherwise).

In order to quantify the instability of IF movement (that is, to quantify impairments in the accuracy, regularities and smoothness), three kinetic parameters were introduced here. First, spatial fluctuation of IF movement was defined as an average of standard deviations of the local maxima (*n* = 10) and those of the local minima (*n* = 10) for each subject (Figure 5Aa–c). Second, to reveal temporal fluctuation of movements of IF, we measured intervals from the nose to the target and from the target to the nose and calculated standard deviations (Figure 5Ad–f). Third, we also calculated the average speeds of their IF-movements (Figures 4Ab,Bb, 5Ag–i) as another parameter to reveal dynamic instability of movements of IF. We statistically compared the patients (*n* = 5) with the control (*n* = 5). Although there was significant difference of the average speed between the two groups (Figure 5Ag; *p* = 0.032, Mann–Whitney *U*-test), there were no significant differences of the spatial fluctuations (Figure 5Aa; *p* = 0.841, Mann–Whitney *U*-test) and the temporal fluctuations (Figure 5Ad; *p* = 0.151, Mann–Whitney *U*-test) between the patients and the controls. When we exclude the patient showing high value, there was no significant differences of the spatial fluctuations (*p* = 0.064, Mann–Whitney *U*-test).

Next, we examined the relationship between these three parameters of the patients and their SARA scores. There were no significant correlation of the special fluctuations (Figure 5Ab; *r*_s_ = 0.64, *p* = 0.216, Spearman's rank correlation), the temporal fluctuations (Figure 5Ae; *r*_s_ = −0.15, *p* = 0.633, Spearman's rank correlation) and the average speed (Figure 5Ah; *r*_s_ = 0.82, *p* = 0.067, Spearman's rank correlation) with SARA score. In addition, the average speed significantly correlated with the scores of the nose-finger test in SARA (Figure 5Ai; *r*_s_ = 0.95, *p* = 0.033, Spearman's rank correlation), while the spatial (Figure 5Ac; *p* = 0.100, Spearman's rank correlation) and temporal (Figure 5Af; *p* = 0.267, Spearman's rank correlation) fluctuations did not significantly correlated with the scores. Overall, the average speed appears to be the most sensitive parameter to reveal ataxia in the upper limb.

Finally, to compare fluctuation of more proximal parts (RE and HD) we calculated an average of standard deviations of horizontal, vertical and depth directions for movements of RE and HD (Figures 5B,C). There were no significant differences of the fluctuations of RE and HD between the controls (*n* = 5) and the patients (*n* = 5). The fluctuations of RE and HD did not significantly correlate with their score of the nose-finger test in SARA (Figures 5Bc, Cc; *P* > 0.05, Spearman's rank correlation). They did not significantly correlate with SARA score (Figure 5Bb; *r*_s_ = 0.61, *p* = 0.15, and Cb; *r*_s_ = 0.82, *p* = 0.067, Spearman's rank correlation).

### The Task of Walking

**Figure 6:**
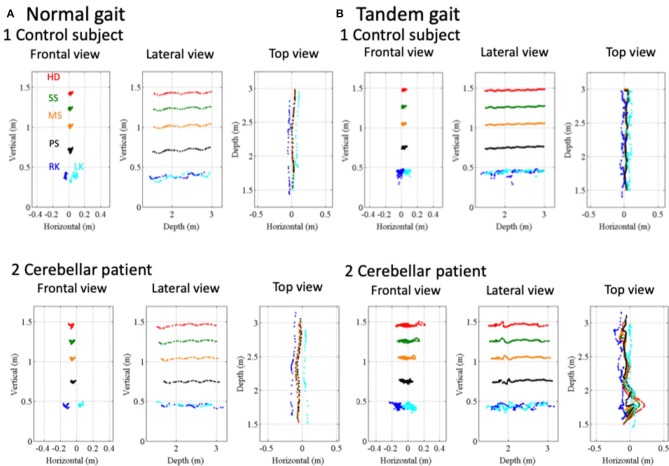
Movement kinematics of reference points in the gait and tandem gait. **(A**1**)** Positions of a control subject's HD (red dots), SS (green dots), PS (black dots), MS (orange dots), RK (blue dots), and LK (cyan dots) in frontal view, lateral view, and top view in the normal gait. **(A**2**)** Positions of HD, SS, MS, PS, RK, and LK of a cerebellar patient in frontal view, lateral view, and top view in the normal gait. **(B**1**)** Positions of the control subject's HD, SS, MS, PS, RK, and LK in frontal view, lateral view, and top view in the tandem gait. **(B**2**)** Positions of the cerebellar patient's HD, SS, MS, PS, RK, and LK in frontal view, lateral view, and top view in the tandem gait.

The control and patient with CCA walked along a 1.5-m walkway with the normal gait (Figure 6A) and with the tandem gait (Figure 6B) approaching to the Kinect v2 sensor. The patient horizontally staggered a little bit at the point 2.35 m in the depth axis from the sensor in the normal gait (Figure 6A2). The horizontal distances between the patient's right knee (RK, blue dots in Figure 6A) and left knee (LK, cyan dots in Figure 6A) were larger than those of the control (Figures 6A1,A2: *p* = 4.00 × 10^−13^, Mann–Whitney *U*-test). In the tandem gait, the horizontal movements of the patient's trunk (SS, MS, and PS) and HD also were larger than those of the control (Figures 6B1,B2: SS, *p* = 1.22 × 10^−32^; MS, *p* = 3.54 × 10^−33^; PS, *p* = 3.23 × 10^−30^; HD, *p* = 8.90 × 10^−19^ by Mann–Whitney *U*-test). Especially, we observed large leftward sway of HD and SS due to a stagger of the patient at 1.77 m from the sensor as clearly seen in the top view (Figure 6B2).

**Figure 7:**
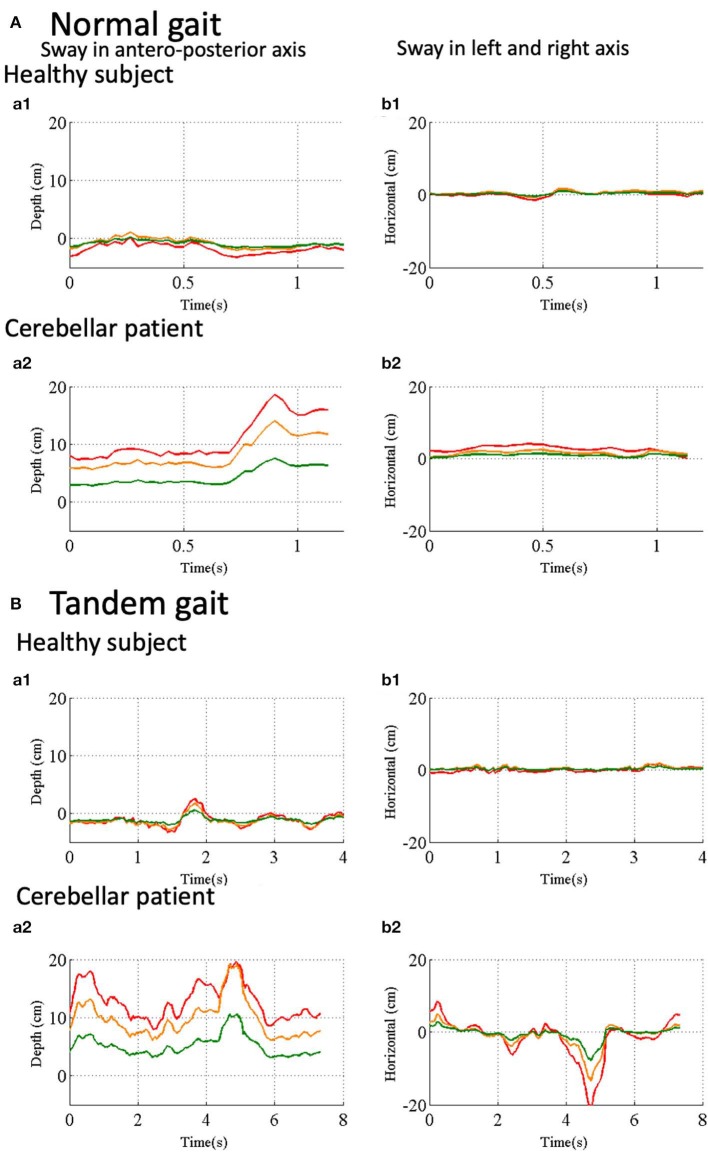
Trunk and head movements relative to the reference point in the pelvis. **(A**a1**)** Sway in antero- posterior direction in normal gait of a control subject. Depth distance of HD (red line), SS (orange line) and SM (green line) from the Kinect v2. **(A**a2**)** Sway in left and right direction in normal gait of the control subject. Horizontal positions of HD (red line), SS (orange line) and SM (green line) in normal gait of the control subject. **(A**b1**)** Sway in antero-posterior axis in normal gait of a cerebellar patient. **(A**b2**)** Sway in left and right axis in normal gait of the same patient. **(B**a1**)** Sway in antero-posterior axis in tandem walk of the control subject. **(B**a2**)** Sway in left-right axis in tandem walk of the control subject. **(B**b1**)** Sway in antero-posterior direction in the tandem walk of the cerebellar patient. **(B**b2**)** Sway in left-right axis in tandem walk of the same patient.

To evaluate fluctuation of trunk, we calculated relative position of three reference points, HD, SS and MS (see Figure 1E) to the pelvis (PS, see Figure 1E). During the normal gait, the control subject showed little deviation from zero, indicating HD, SS, MS, and PS are all aligned vertically (Figure 7Aa1,b1). In the patient, however, HD was deviated anteriorly by about 8 cm at the beginning and the deviation peaked around 0.8 s after the start (Figure 7Aa2), with little left-right deviation (Figure 7Ab2), indicating that the patient walked in a forward-bent posture. In the tandem gate, although the control subject showed little left or right deviation from zero (Figure 7Bb1), she showed a small stagger anteriorly (Figure 7Ba1). On the other hand, the patient showed not only the marked anterior deviation but also a large stagger for antero-left direction (Figures 7Ba2,Bb2), indicating ataxic stagger in the tandem gait.

**Figure 8:**
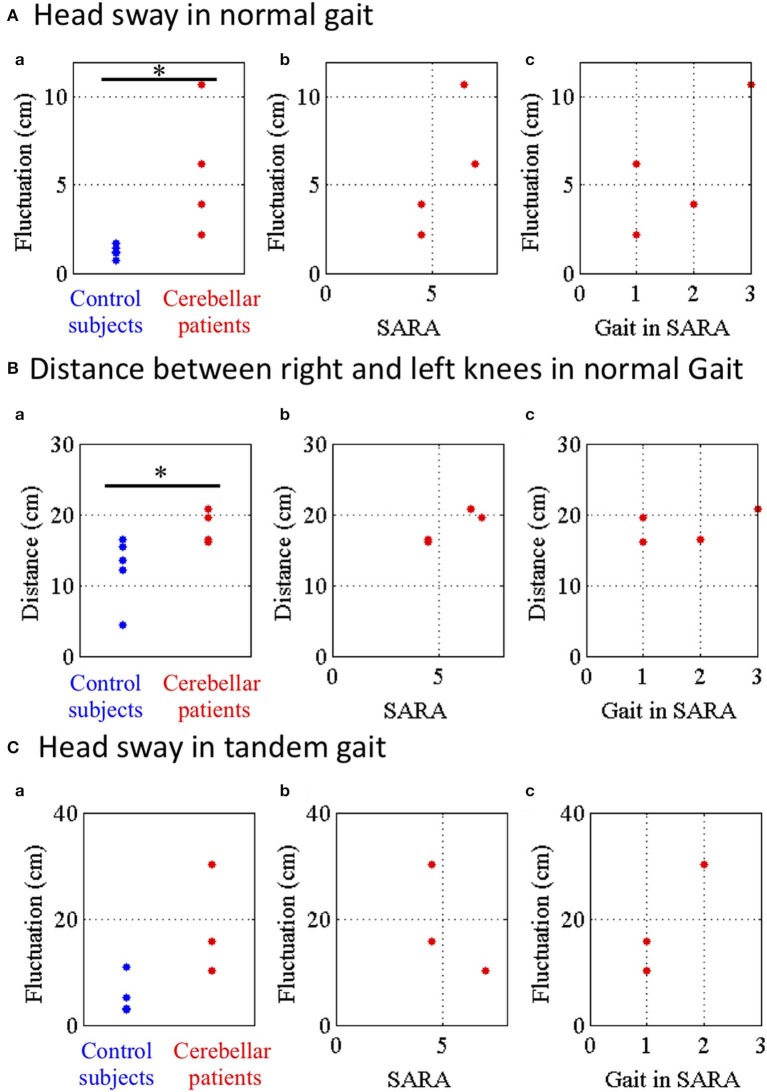
Parameters of head sway in normal and tandem gaits. **(A)** Head sway representing horizontal and depth deviation of HD relative to PS in normal gait. (a) Blue and red dots show fluctuations of control subjects and cerebellar patients in normal gait. (b) Relationship between the fluctuations and SARA or (c) score of the nose-finger test in SARA**. (B)** Distance between RE and LE in normal gait. **(C)** Head sway in tandem walk. *p < 0.05 by Mann–Whitney U test.

To evaluate instability of movements of the head during the gaits, fluctuations of the head in regular (Figure 8A) or tandem (Figure 8C) gaits were defined as horizontal and depth deviation of HD relative to PS during walking. Moreover, to evaluate wide-based walk, we measured the horizontal distance between RK and LK in the normal gait (Figure 8B). We compared fluctuations of head sway of the controls (*n* = 5) and those of the patients (*n* = 4) except for a patient in the normal gait because the patient was unable to stand alone. The head sway of the patients was significantly larger than those of the controls (Figure 8Aa; *p* = 0.032, Mann–Whitney *U*–test). Nevertheless, it did not significantly correlate with either SARA score (Figure 8Ab; *p* = 0.500, Spearman's rank correlation) or a score of the gait task in SARA (Figure 8Ac; *p* = 0.426, Spearman's rank correlation). We found that the inter-knee distances of the patients were significantly larger than those of the controls (Figure 8Ba; *p* = 0.032, Mann–Whitney *U*-test). However, it did not significantly correlate with either SARA score (Figure 8Bb; *p* = 0.167, Spearman's rank correlation) or a score of the gait task in SARA (Figure 8Bc; *p* = 0.250, Spearman's rank correlation). This suggests that instability of the normal gait was at least partly compensated by spreading their legs in case of mild ataxia.

Finally, we compared the fluctuations of head sway of the controls with those of the patients except for two patients in the tandem gait because they were unable to stand alone stably in tandem. Although the fluctuations of the patients were larger than those of the controls, there was no significant difference of the fluctuations between the controls and the patients (Figure 8Ca; *p* = 0.143, Mann–Whitney *U*-test). Furthermore, the fluctuations did not significantly correlate with either SARA score (Figure 8Cb; *p* = 1.00, Spearman's rank correlation) or a score of the gait task in SARA (Figure 8Cc; *p* = 0.333, Spearman's rank correlation). These findings suggest that cerebellar patients walked with larger sways of their trunk in the normal gait as well as in the tandem gait.

## Discussion

We developed a device analyzing CAs with a precision within <2 mm. We designed our device to maintain consistency with SARA and to improve the accuracy of SARA by ~10 times. For instance, our device detected scattered trajectory in nose-finger test and instability of trunk in walking task. In addition, our device collects much more information than SARA by simultaneously recording 25 points on the whole body. Because of that, our device highlighted instability of head, elbow and trunk in nose-finger test and increased inter-knee distance in walking task, which are not described in the instruction of SARA.

### Quantitative Evaluation of Movement Kinematics in Previous Studies

There are a number of studies that tried to quantify ataxic movements experimentally [e.g., (5–9, 11, 12, 18)]. Each study confined its point of evaluation to only one part of the body, such as the forelimb [e.g., (5–8, 12, 19)] or gait [e.g., (9–11)]. Since these studies were not designed to make comprehensive assessments of CAs like SARA, they were not compatible with SARA. More recently, Krishna et al. (20) employed three tests for movements of upper and lower limbs, i.e., nose-finger test, diadochokinesis test and heel to shin test, and tried to evaluate CAs. However, each test measured linear acceleration and angular velocity at one part of the body and has focused on their regularity (20). In other words, they evaluated parameters that were not compatible with SARA. On the other hand, we tried to reproduce two tests of SARA as compatible as possible with Kinect v2. In addition to the reproducibility, our device is characterized by measuring simultaneously 25 reference points on the whole body during a task without markers or sensors on participant's body, which allows us to extract more information from CAs than SARA (Table 1).

**Table 1:** Classification of measurements in digitalized SARA.

	**Ataxic outcome** **(e.g., Clumsiness, irregularity and inaccuracy in requested movements)**	
	**Evaluated in SARA**	**Not evaluated in SARA**
Nose-finger test	Scattered trajectory of index finger - Increased fluctuations in the trajectory distance - Increased fluctuations in the interval of each trial - Average speed*	Instability of head, elbow, and trunk
Walking task	Instability of trunk - Large sways of spine and head*	Increased inter-knee distance

*The items indicated by asterisk were statistically significant*.

### Reproducibility and Multiplicity in Digitalized SARA

The results of this pilot study showed that our device using Kinect v2 accurately measured ataxic movements employed in SARA (Table 1) with high accuracy of better than 2 mm by simple comparison with a ruler. On the other hand, previous studies that validated accuracy of Kinect used other motion captures such as *CMS20s* (Zebris, Germany) for forelimb movement of stroke patients (19) or *Optotrak Certus System* (Northern Digital, Canada) for gait of healthy controls (21). However, they were not able to provide absolute accuracy in the measurement of Kinect. Overall, our device simply improves the accuracy of SARA by ~10 times. Therefore, it is straightforward for clinicians to translate outputs of our device into scores of SARA. In addition, other aspects of the ataxic movements, which are not evaluated in SARA, were also captured by simultaneously recording 25 reference points of the whole body with our device (Table 1). For instance, in nose-finger test, it recorded entire trajectories of ataxic movement of IF precisely. Furthermore, it also captured adventitious movements (i.e., instability) of the trunk, shoulder and elbow, suggesting that nose-finger test is not a pure evaluation of arm movement, rather it also evaluates stability of the trunk simultaneously. In the walking task, it captured instability of not only of the lumbar position, which is itemized in SARA (3), but also that of head and upper trunk separately. In addition, our device also quantifies an increase in distance between knees, compensations of the unstable large sways. Overall, our device is capable to quantifies various parameters that define the unstable walking, from the large sway to the compensation, with higher accuracy than SARA. The results suggest that a number of aspects of CAs, such as clumsiness, irregularity, inaccuracy and instability, can be simultaneously identified and quantified with our digitalized SARA without increasing efforts of patients. It should be acknowledged that some of these divergent ataxic features are overlooked in ordinary clinical examinations. It should be noted that our device is easily applicable for evaluation of kinematics of other neurological disorders such as stroke, Parkinson's disease or dystonia.

### Ataxic Outcomes and Ataxic Elementary Symptom

Importantly, our device also identified distortion in more elementary processes (*ataxic elementary symptom*) causing or underlying the *ataxic outcome*, clumsiness, irregularity and inaccuracy in a requested movement. The ataxic elementary symptom would be more directly linked to disorganized behavior due to the cerebellum in motor controls. For example, in the task of nose-finger test, our device did not only show scattered trajectories but also adventitious movements in the proximal joints, which reflect a disorganized Purkinje cell (PC)-mediated inhibition of the dentate nucleus (DN), an important disorder at a neural circuitry level (22). Importantly, the degree of scattering can be also quantified.

In the cerebellar circuitry, inputs conveyed by mossy fibers are transmitted through two modes; (1) granule cells (GC) and parallel fibers (PF)-PC and (2) GC and PF-Basket cells (BC)-PC (23). The former pathway activates PC, resulting in inhibition of neurons in the deep cerebellar nuclei (DCN), whereas the latter pathway suppresses PC, resulting in excitation of the DCN neurons. This means that temporal patterns of outputs from the cerebellum are controlled by switching two modes in the context of motor control (inhibition/disinhibition theory) (22). It is presumed that impairment of the inhibition causes recruitment of excessive muscle activities, whereas that of the disinhibition causes delays in motor initiation or slowness in attaining exertion upon full power. Consistent with this assumption, Holmes (1) described these elementary symptoms as adventitious movements and asthenia, respectively (22). Although these elementary symptoms are easily overlooked in daily neurological examinations, our device clearly identified the adventitious movements in the task of the nose-finger test. Thus, our results suggest that with our device, clinicians can identify the elementary symptoms that are more directly linked to disorganized cerebellar motor control. This is a distinguishable feature in our Kinect system when compared with other objective examination devices which have been developed so far.

In conclusion, our new measurement device is composed of (1) the Kinect, which captures the position of the reference points and (2) the new algorithm, which converts the position data to parameters that can be assessed by SARA. Our device comprehensively captured the ataxic outcome in terms of both at the focus of attention of neurologists and in its surroundings in evaluation of the nose-to finger and walking tasks. Furthermore, the device identified more extensive aspects of ataxic outcomes in each task of SARA and uncovered more elementary symptoms that could be explained physiologically. Once these elementary symptoms are recognized among specialists, they can contribute to better assessment of grading of ataxia. In other words, quantitative device which comprehensively characterizes elementary disorders underlying ataxic outcomes will be beneficial. It is also possible to separate change in CA symptoms and physiological fluctuations, by repeating measurements in short intervals of time.

Further studies are necessary to digitalize the other tasks of SARA and to develop algorithms that can further quantify the present measurements. Finally, the use of fully-digitalized SARA at bedside will help even for non-expert examiners to make more reliable evaluation of SARA. Such a device provides an ideal platform to track various aspects of motor functions of cerebellar patients for a longer period and in an unprecedentedly larger scale. It will, in turn, provide us a deeper functional interpretation for each test of SARA. It should be also emphasized that a large scale dataset provided by such a device is ready for analysis with AI. We are currently working on development of such a device.

## Conclusions

We have developed a device that captures comprehensively ataxic movements of patients with cerebellar diseases with a precision of <2 mm. Our device not only reproduced but also improved the clinical evaluation of neurologists. Our device further highlighted other aspects of ataxic movements that are not defined in the instruction of SARA. The present study demonstrated a potential of fully-digitalized SARA for tracking progression of cerebellar dysfunctions and future development of treatments for spinocerebellar degeneration.

## Data Availability Statement

Deidentified participant data will be shared, as well as the study protocol and statistical analyses, upon reasonable requests.

## Ethics Statement

All experiments were performed in accordance with relevant guidelines and regulations. The experimental protocol was approved by the Ethics Committee of Tokyo Medical University (2017-035), National Center of Neurology and Psychiatry (A2018-104) and Tokyo Metropolitan Institute of Medical Science (18–41). All participants were informed about the research procedure and signed a written consent form.

## Author Contributions

SKa, THo, and HMit conceived and designed the research. THo, HY, SY, and TK developed a device. HT, TT, HA, YM, SKu, KB, THa, and HMiz enrolled participants and provided clinical supports. THo, HY, YM, and KB performed the experiments. THo, SKa, and HMit analyzed data. THo, SK, HMit, MM, THa, HMiz, HA, TK, and SKu. wrote the manuscript. All authors read and approved the final manuscript.

### Conflict of Interest

The authors declare that the research was conducted in the absence of any commercial or financial relationships that could be construed as a potential conflict of interest. The handling editor declared a past co-authorship with one of the authors MM.

## Abbreviations

SARA: Scale for the Assessment and Rating of Ataxia
SCA 6: spinocerebellar ataxia type 6
CCA: cortical cerebellar atrophy
MSA-C: multiple system atrophy, cerebellar type
IF: right index finger
RS: right shoulder
LS: left shoulder
RE: right elbow
LE: left elbow
RK: right knee
LK: left knee
HD: head
SS: a middle point of their left and right shoulders on their spine
PS: pelvis on their spine
MS: a middle point of SS and PS
Tr: Trunk consists of SS, PS and MS
NE: neck
RA: right ankle
LA: left ankle.
